# Supplementary material for: A complex between IF2 and NusA suggests early coupling of transcription-translation
Source: Nat Commun. 2025 Jul 26;16:6906. doi: 10.1038/s41467-025-62207-w (PMC12297416; doi:10.1038/s41467-025-62207-w)
Supplement: Supplementary file 1 — Supplementary Information [file 41467_2025_62207_MOESM1_ESM.pdf]

SUPPLEMENTARY MATERIAL

A complex between IF2 and NusA suggests early coupling of transcription-translation

Mikhail Metelev, Magnus Johansson

Department of Cell & Molecular Biology, Uppsala University

Correspondence: m.johansson@icm.uu.se

Table of content

Supplementary Note 1. Impact of HaloTag fusion on the functionality of initiation factors 3

Supplementary Fig. 1. Effect of N-terminal HaloTag fusion on activity of IFs. 4

Supplementary Fig. 2. Step length distributions within diffusion trajectories of labeled factors. 5

Supplementary Fig. 3. Trajectory length distributions of IFs. 6

Supplementary Fig. 4. State transitions in HaloTag-IFs fitted to 2-state models. 6

Supplementary Fig. 5. Fitting of histograms of step length distributions within diffusion trajectories of HaloTag labeled 30S. 7

Supplementary Fig. 6 AIC values for HMM models of 30S, HaloTag, fMet-tRNAfMet, and all labeled IFs. . 8

Supplementary Fig. 7. Fitted HMM models of diffusion states for 30S, HaloTag, fMet-$tRNA^{fMet}$, and all labeled IFs. 9

Supplementary Fig. 8. Coarse-grained results of HMM modelling for HaloTag-labelled IFs and fMet-[Cy5]$tRNA^{fMet}$... 10

Supplementary Fig. 9. Binding kinetics of IFs and fMet-$tRNA^{fMet}$ from 3-state coarse-grained HMM models of sizes 5-9. a, b (related to Fig 2 a-b). 11

Supplementary Note 2. Calculations for the average cycle time of the ribosome to translate a typical protein... 12

Supplementary Fig. 10. Effect of mutation on slow diffusion state occupancy of HaloTag-IF2 and HaloTag-IF3. 13

Supplementary Fig. 11. Fluxes of HaloTag-IF2α particles between states in HMM-fitted 5-state and 9-state models, coarse-grained to 3 or 4 diffusion states. 14

Supplementary Fig. 12 (related to Fig. 2c). Estimated dwell-times of HaloTag labeled IF3 as well as IF2 isoforms and mutants in the “bound” diffusion state from 3-state coarse-grained HMM models of sizes 5-9. 15

Supplementary Fig. 13. Spatial distribution of the slow diffusion state for HaloTag-IF2α and HaloTag-IF2γ isoforms. 16

Supplementary Fig. 14 (related to Fig. 3). Spatial distribution of NusA-HaloTag and RbfA-HaloTag in the fast diffusion state. 17

Supplementary Fig. 15. SDS-PAGE analysis of cell lysates and affinity purification fractions for the presence of HaloTag. 17

Supplementary Fig. 16. Effect of NTD deletion on slow diffusion state occupancy of NusA-HaloTag in WT and in E. coli ΔIF2-Domain-I. 18

Supplementary Fig. 17. Interaction between NusA and IF2α in the bacterial cytoplasm. 19

Supplementary Fig. 18. Modelling of a coupled transcription-translation complex with fitted IF2. 20

Supplementary Note 1. Impact of HaloTag fusion on the functionality of initiation factors

To explore if the addition of a HaloTag compromises IF activity we constructed plasmids for IPTG-inducible expression of individual non-labeled IFs, as well as N-terminal HaloTag fusions of each IF. To test whether there is any growth defect associated with the addition of the HaloTag, the plasmids were introduced in *E. coli* strains in which the corresponding IFs were deleted from the genome (Supplementary Fig. 1). Without IF induction, all strains showed poor growth, suggesting that leakage level expression is insufficient. When IPTG was present in the media at 32-64 µM, the strains with plasmid-expressed IF2α, IF3, and IF1 showed doubling times similar to that of the wild-type (WT) strain carrying an empty plasmid, suggesting that at this induction level, the plasmids support fast growth (Supplementary Fig. 1a,b,d). Higher expression of IF3 inhibited growth, suggesting that proper cell physiology requires a fine-tuned concentration of this factor, in line with in vitro results showing inhibition of translation initiation at high IF3 levels (Supplementary Fig. 1d) [1]. Expression of the IF2γ isoform also supported growth, albeit at a slower rate even at higher concentrations of the inducer (Supplementary Fig. 1c).

For neither of the IF2 isoforms, the addition of HaloTag at the N-terminus altered the growth rate, indicating that HaloTag does not interfere with the factors' functions (Supplementary Fig. 1b,c). Induction of the HaloTag-IF3 fusion could also support fast growth of the strain lacking chromosomal IF3, albeit at a slightly higher concentration of the inducer than needed for the non-HaloTag version (Supplementary Fig. 1d). This indicates that the addition of the HaloTag might have a slight negative effect on IF3 function, but the discrepancy could also be due to changes in expression level or folding efficiency because of the HaloTag addition. Finally, the strain lacking IF1 and expressing HaloTag-IF1 shows noticeable growth defect in comparison with the strain where WT IF1 is expressed from a plasmid at any level of induction, showing that the HaloTag negatively affects the functionality of IF1 (Supplementary Fig. 1a).

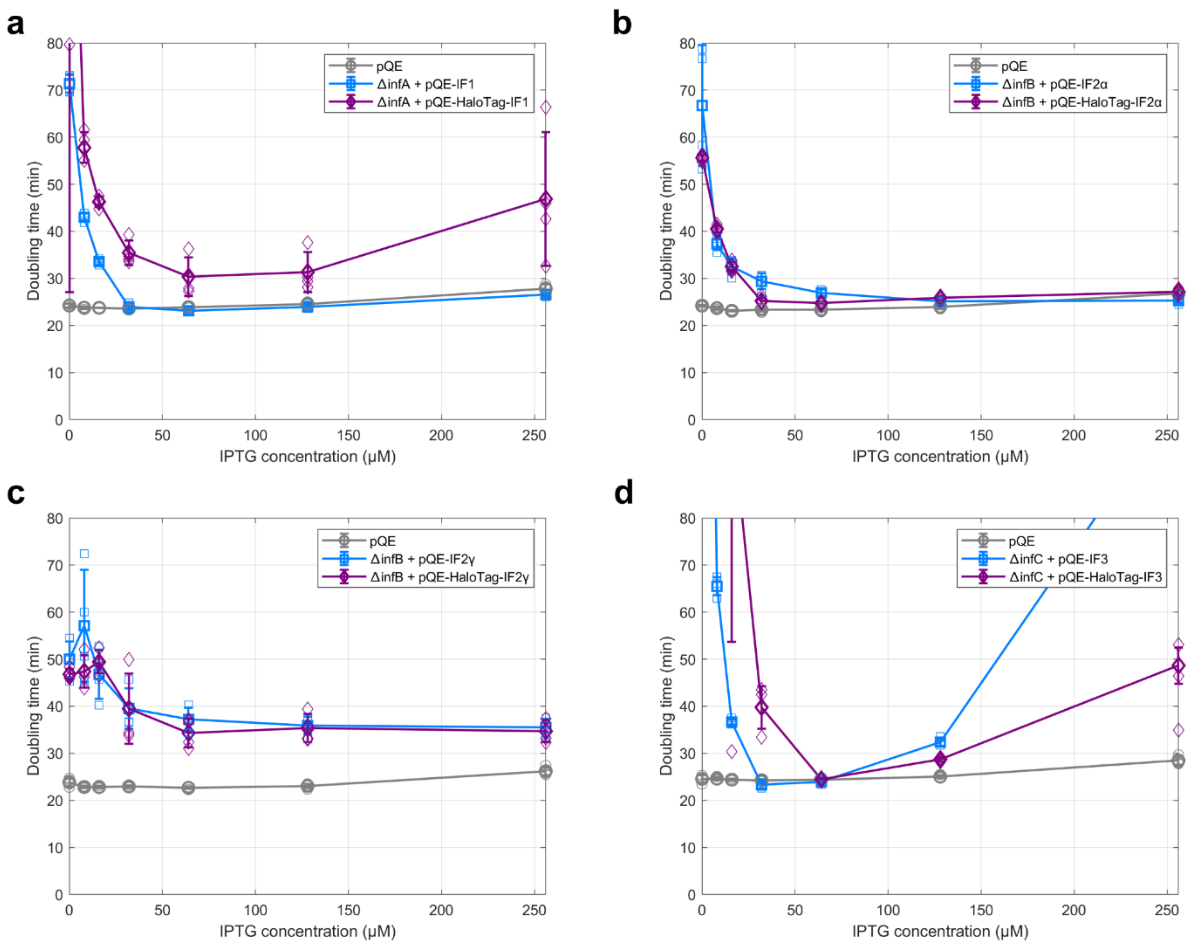

Supplementary Fig. 1. Effect of N-terminal HaloTag fusion on activity of IFs. a. Effect of IPTG concentration on doubling time of *E. coli* strain carrying an empty pQE plasmid and *E. coli ΔinfA* strain carrying either a plasmid pQE-IF1 expressing IF1 protein or a plasmid pQE-HaloTag-IF1 expressing HaloTag-IF1 fusion. b. Effect of IPTG concentration on doubling time of *E. coli* strain carrying an empty pQE plasmid and *E. coli ΔinfB* strain carrying either a plasmid pQE-IF2α expressing α isoform of IF2 protein or a plasmid pQE-HaloTag-IF2α expressing HaloTag-IF2α fusion protein. c. Effect of IPTG concentration on doubling time of *E. coli* strain carrying an empty pQE plasmid and *E. coli ΔinfB* strain carrying either a plasmid pQE-IF2γ expressing γ isoform of IF2 protein or a plasmid pQE-HaloTag-IF2γ expressing HaloTag-IF2γ fusion protein. d. Effect of IPTG concentration on doubling time of *E. coli* strain carrying an empty pQE plasmid and *E. coli ΔinfC* strain carrying either a plasmid pQE-IF3 expressing IF3 protein or a plasmid pQE-HaloTag-IF3 expressing HaloTag-IF3 fusion. Source data are provided as a Source Data file.

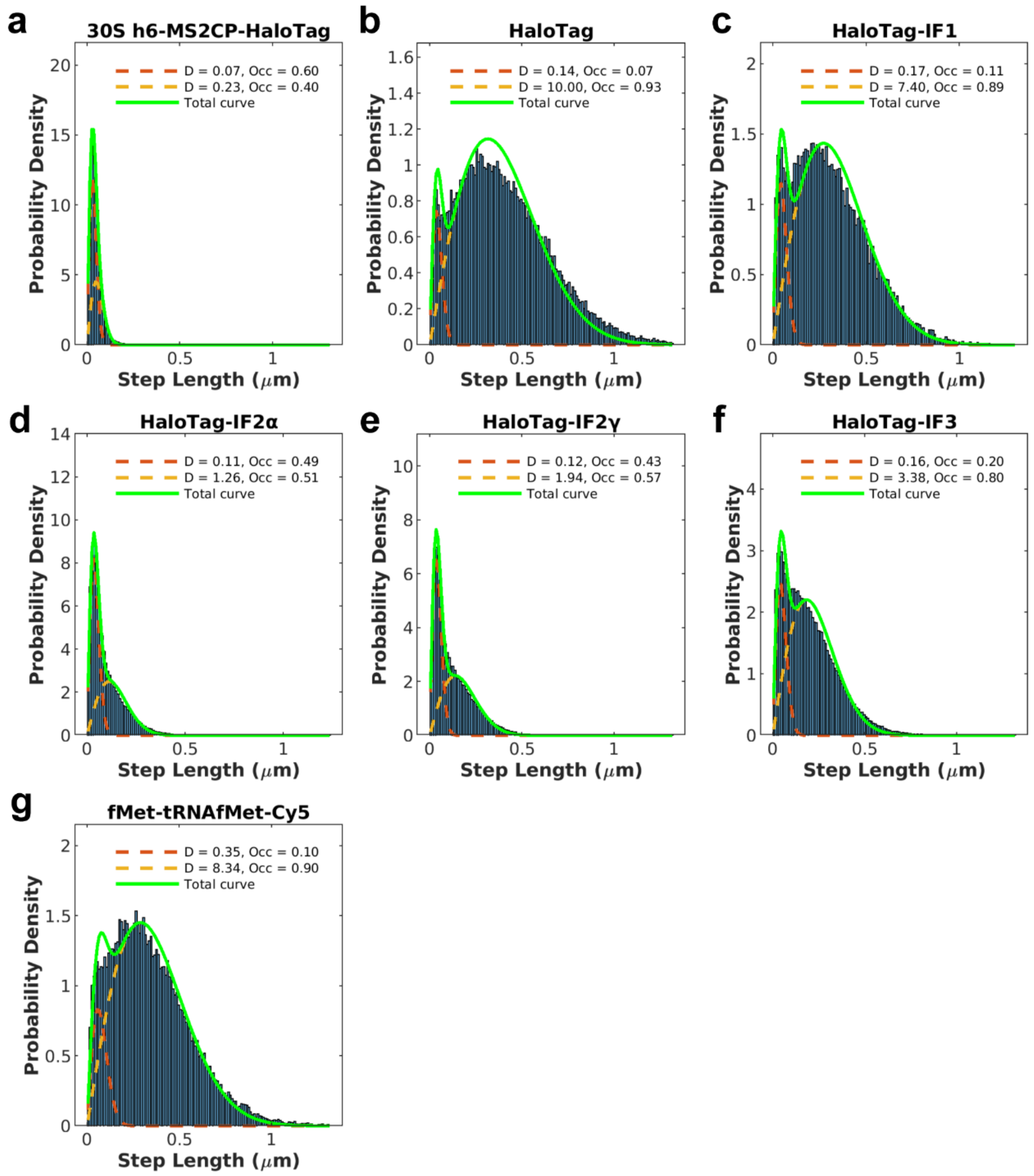

Supplementary Fig. 2. Step length distributions within diffusion trajectories of labeled factors. Histograms of step length distributions collected for combined datasets for (**a**) HaloTag labeled 30S subunits (324164 steps in 7063 trajectories), (**b**) HaloTag (109253 steps in 7795 trajectories), (**c**) HaloTag-IF1 (143967 steps in 7444 trajectories), (**d**) HaloTag-IF2α (134697 steps in 3848 trajectories), (**e**) HaloTag-IF2γ (127210 steps in 4051 trajectories), (**f**) HaloTag-IF3 (172989 steps in 6500 trajectories), (**g**) fMet-tRNA^{fMet}-Cy5 (53849 steps in 3033 trajectories). Histograms were fitted with a combination of two gamma distributions. The fits show that HaloTag-IFs and fMet-tRNA^{fMet}-Cy5 exhibit a mixture of diffusion states, with one major slow state (similar to the 30S subunit) and a factor-specific fast state. D – diffusion coefficient of the fitted state; Occ – occupancy in the corresponding state. Source data are provided as a Source Data file.

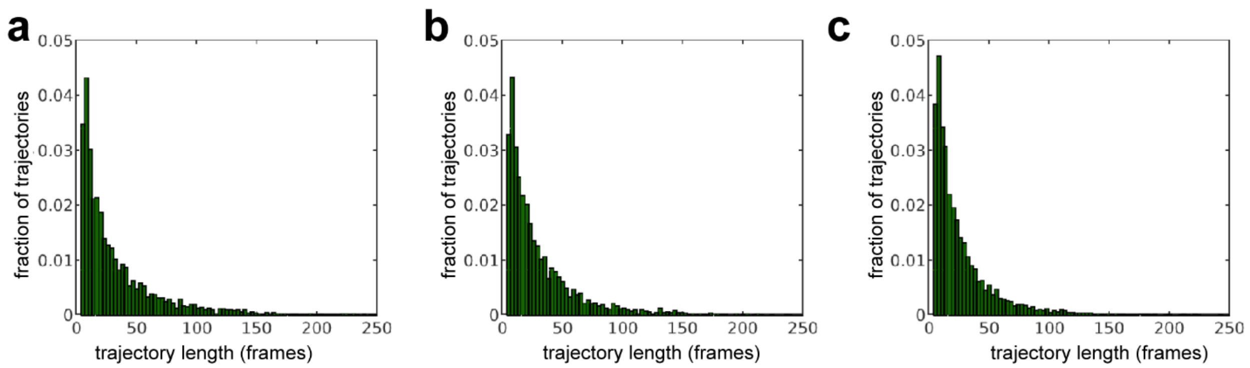

Supplementary Fig. 3. Trajectory length distributions of IFs. Trajectory length distributions for plasmid expressed HaloTag-IF2α (**a**), Halo-IF2γ (**a**), and IF3 (**c**) tracked with 5 ms camera exposure time. Source data are provided as a Source Data file.

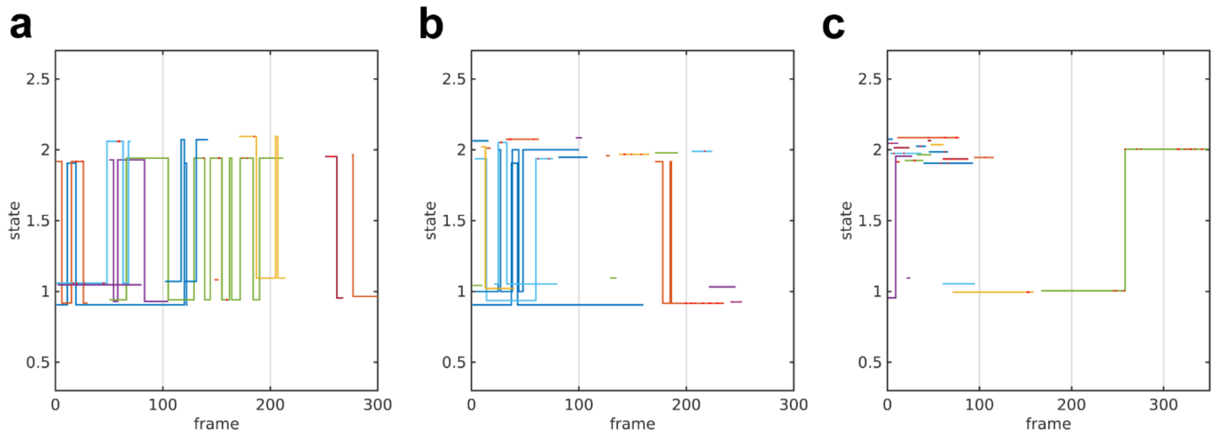

Supplementary Fig. 4. State transitions in HaloTag-IFs fitted to 2-state models. HMM fitted state transitions in diffusion trajectories detected in Supplementary Movies 2 (panel **a**, HaloTag-IF2α), 3 (panel **b**, HaloTag-IFγ), and 4 (panel **c**, HaloTag-IF3).

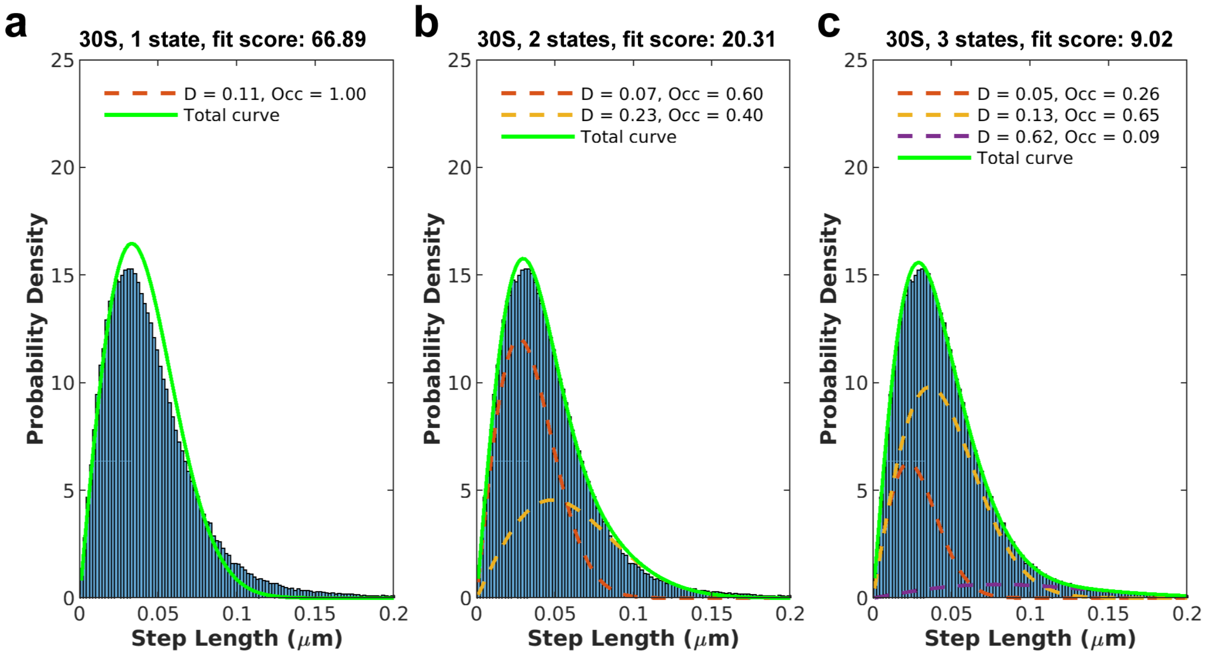

Supplementary Fig. 5. Fitting of histograms of step length distributions within diffusion trajectories of HaloTag labeled 30S. Histograms of step length distributions collected for HaloTag labeled 30S subunits (324164 steps in 7063 trajectories) were fitted with a combination of one (**a**), two (**b**), or three (**c**) gamma distributions. The fit score represents the sum of absolute errors between the empirical step length histogram and the predicted multi-state diffusion model distribution. Source data are provided as a Source Data file.

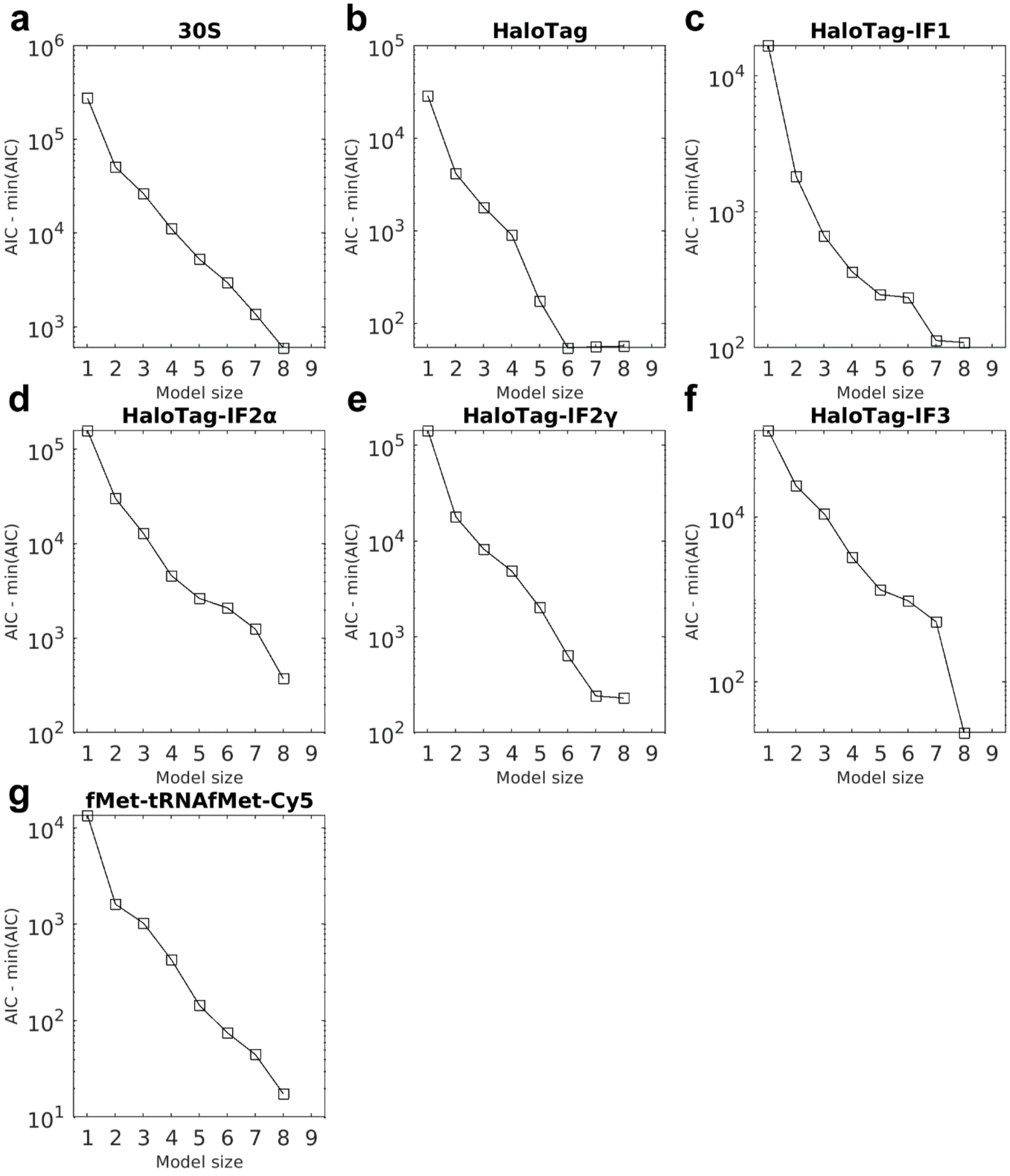

Supplementary Fig. 6. AIC values for HMM models of 30S, HaloTag, fMet-tRNA^{fMet}, and all labeled IFs. In all datasets, the lowest AIC values (i.e., min(AIC)) were obtained with the most complex HMM model tested, containing 9 diffusion states. Source data are provided as a Source Data file.

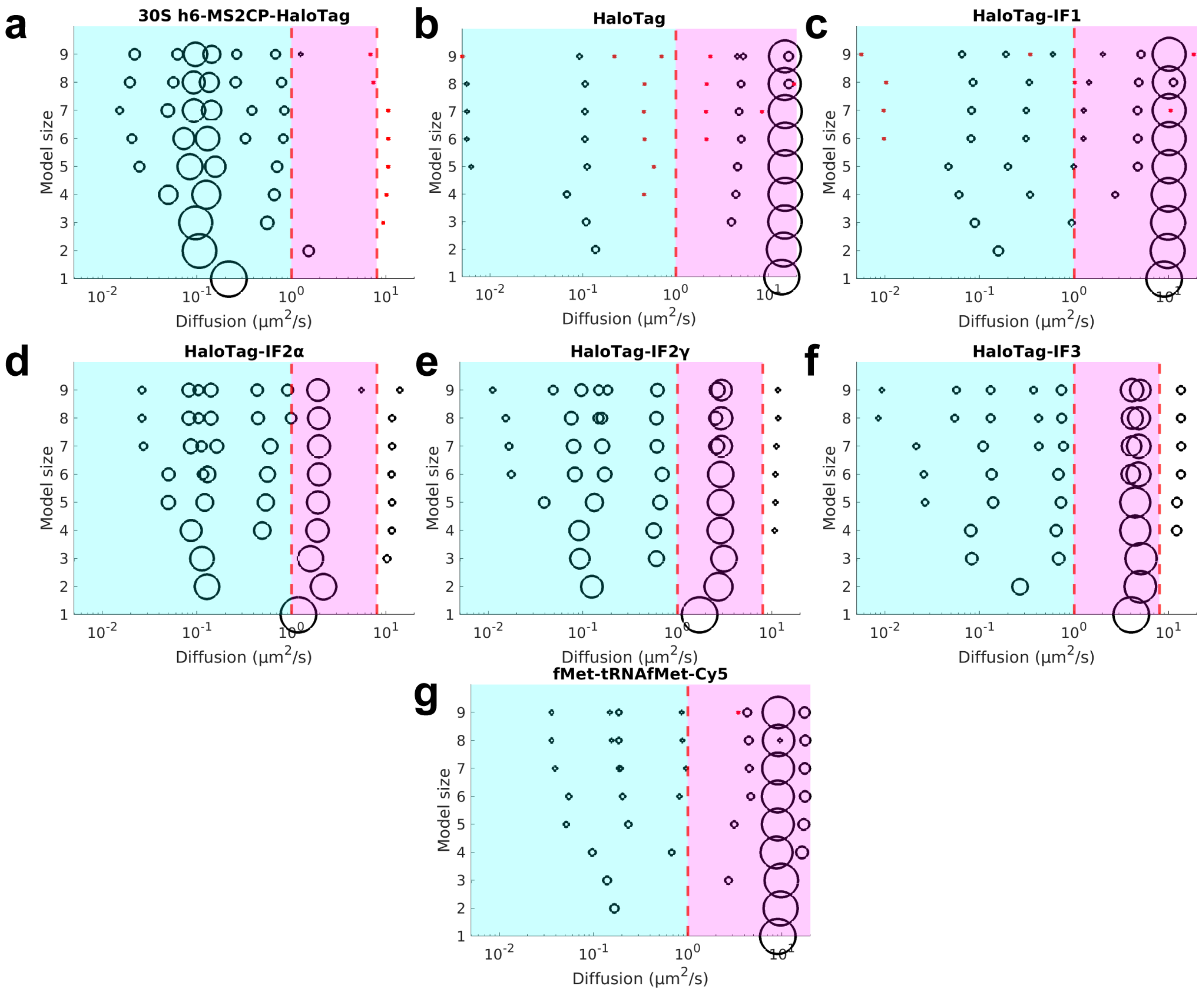

Supplementary Fig. 7. Fitted HMM models of diffusion states for 30S, HaloTag, fMet-tRNA^{fMet}, and all labeled IFs. The area of the circles represents the relative occupancy in different diffusion states. Diffusion states with occupancies lower than 1% are marked as *. Red dashed lines indicate thresholds at 1 μm²/s and 8 μm²/s, used to distinguish ribosome-bound states (cyan area) from free factors (magenta area) and cleavage products. A distinct population of cleavage products was identified only in the datasets for HaloTag-IF2α, HaloTag-IF2γ, HaloTag-IF3, and 30S labeled via h6-MS2CP-HaloTag. The data show results from HMM models for combined datasets. The combined number of cells and trajectory steps were as follows: 30S h6-MS2CP-HaloTag – 2,732 cells and 324,164 steps in 3 independent experiments; HaloTag – 2,630 cells and 109,253 steps in 4 independent experiments; HaloTag-IF1 – 2,586 cells and 143,967 steps in 5 independent experiments; HaloTag-IF2α – 1,592 cells and 134,697 steps in 4 independent experiments; HaloTag-IF2γ – 1,622 cells and 127,210 steps in 5 independent experiments; and HaloTag-IF3 – 2,491 cells and 172,989 steps in 6 independent experiments; fMet-tRNA^{fMet} – 2,039 cells and 53,849 steps in 3 independent experiments. Results for all model sizes for combined data are provided in Supplementary Data 1-7. Source data are provided as a Source Data file.

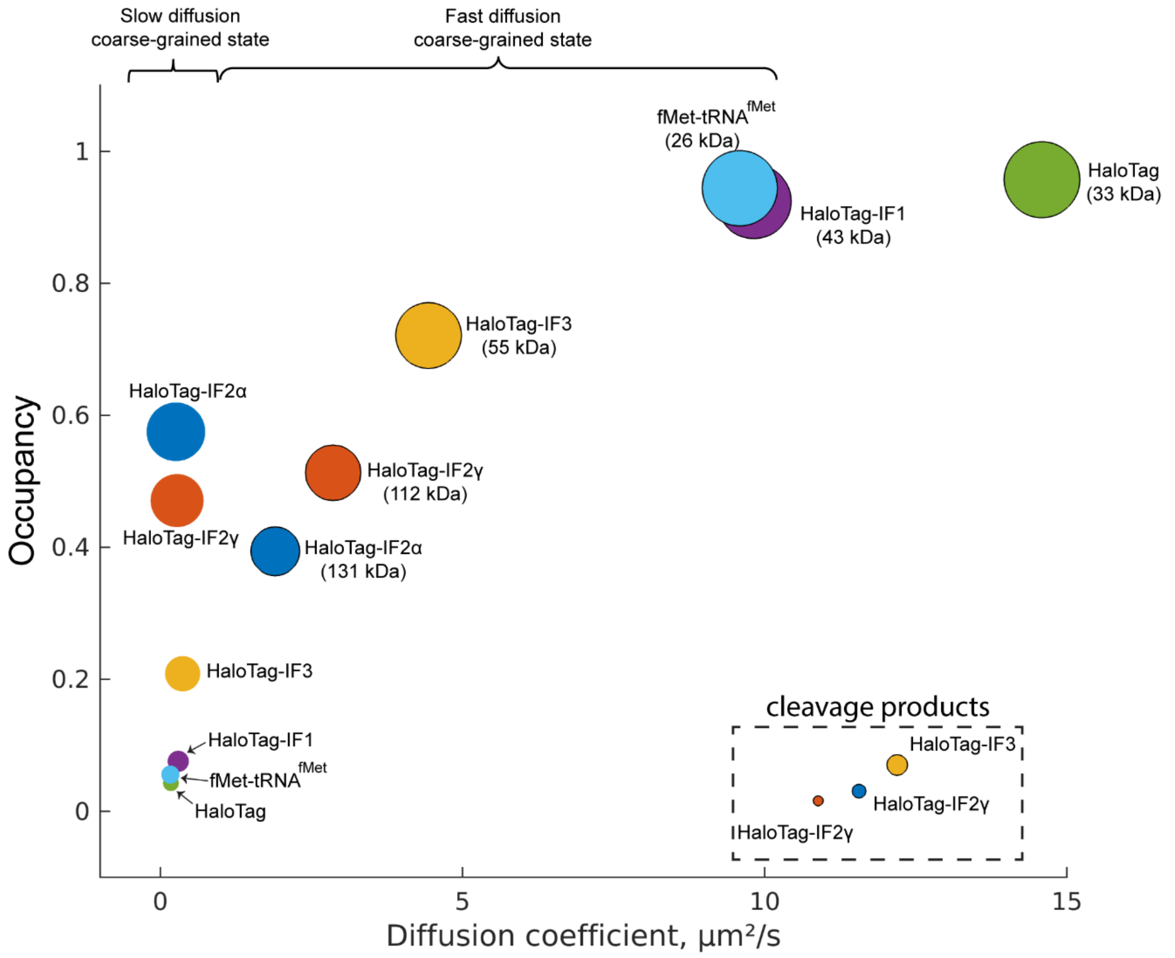

Supplementary Fig. 8. Coarse-grained results of HMM modelling for HaloTag-labelled IFs and fMet-[Cy5]tRNA^{fMet}. Estimated steady-state occupancies of HaloTag-IF2α, HaloTag-IF2γ, HaloTag-IF3, and fMet-[Cy5]tRNA^{fMet} in the slow and fast diffusion states, as well as cleavage products for HaloTag-IF2α, HaloTag-IF2γ, HaloTag-IF3 after coarse-graining from 5-state HMM models (Supplementary Data 8-13). The area of the circles represents the relative occupancy in a coarse-grained diffusion state. Source data are provided as a Source Data file.

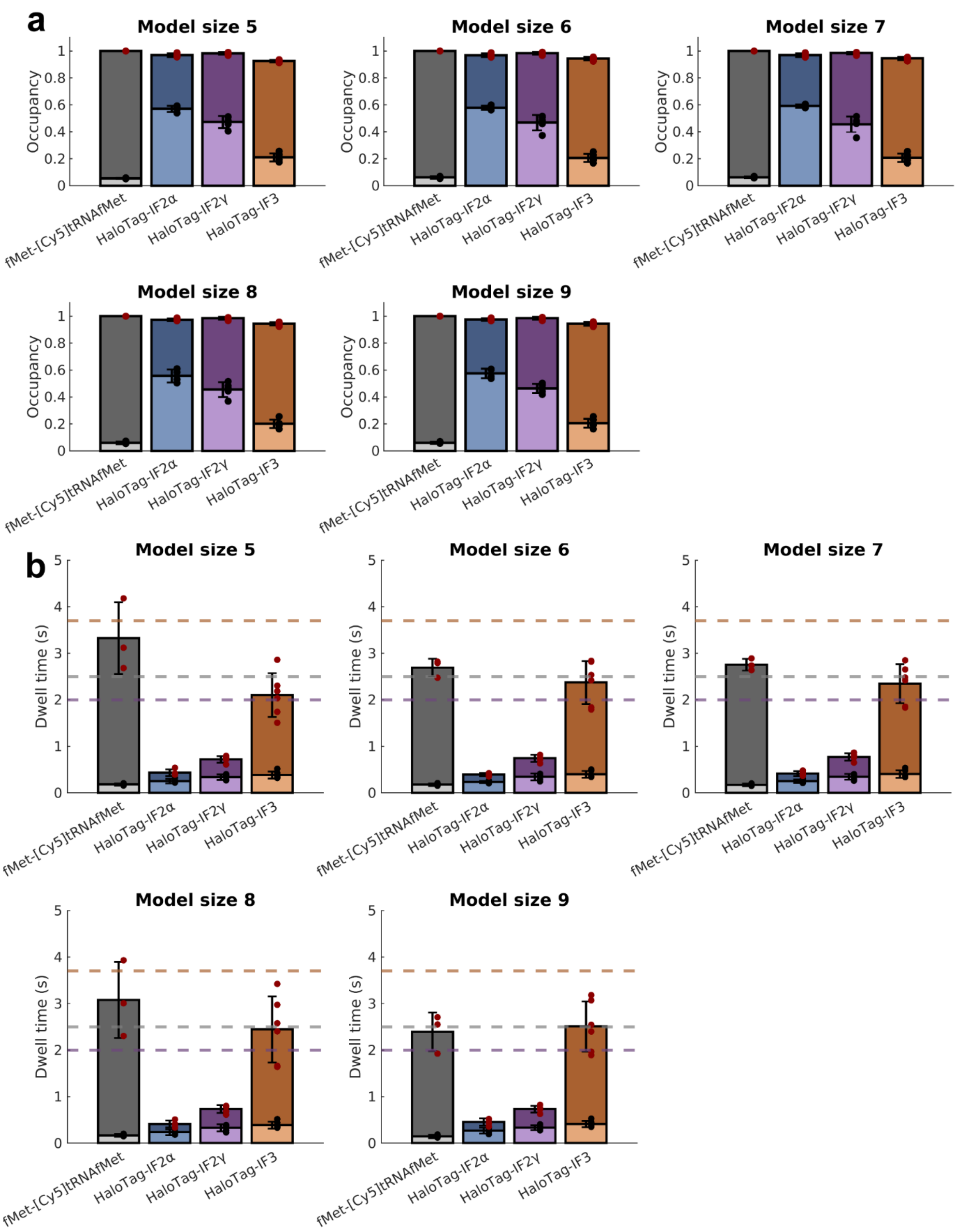

Supplementary Fig. 9. Binding kinetics of IFs and fMet-tRNA^{fMet} from 3-state coarse-grained HMM models of sizes 5-9. a, b (related to Fig 2 a-b). Estimated occupancies (**a**) and dwell-times (**b**) of labeled factors in the "bound" (bottom bar) and the "free" (top bar) diffusion states. Dashed lines in (**b**) are thresholds for estimates of

the theoretical expected total time for a single initiation event. The violet, grey and orange dashed lines indicate the thresholds at 2.0 s, 2.5, and 3.7 seconds, for IF2, fMet-$tRNA^{fMet}$, and IF3, respectively. The data show coarse-grained results from 5-9 state HMM models, with averages calculated from independent experiments. Error bars represent standard deviations between these independent experiments. The combined number of cells and trajectory steps were as follows: fMet-$tRNA^{fMet}$ – 2,039 cells and 53,849 steps in 3 independent experiments; HaloTag-IF2α – 1,592 cells and 134,697 steps in 4 independent experiments; HaloTag-IF2γ – 1,622 cells and 127,210 steps in 5 independent experiments; and HaloTag-IF3 – 2,491 cells and 172,989 steps in 6 independent experiments. Source data are provided as a Source Data file.

Supplementary Note 2. Calculations for the average cycle time of the ribosome to translate a typical protein

Elongation rate of the ribosome at a growth rate of 2 doublings/h (matching the growth rate of the strains in microscopy experiments) has been estimated to be 16-17 amino acids per second in studies where direct measurements of the elongation rate were performed [2]. Indirect calculations based on the amount of total protein per cell, the number of ribosomes per cell, the percentage of active ribosomes (set at 85%), and the cell growth rate, provide an estimate for the global elongation rate at 21-22 amino acid per second [3].

Estimates for the average length of a typical *E. coli* protein varies between different approaches, where ribosome profiling data provide an estimate at 192-222 amino acids [4] (see Supplementary Data 15 in [5]), and estimation based on proteomics data provides an estimate at 242 amino acids [6] (see Supplementary Data 14 in [5]). Taking these estimates into account, elongation of a 'typical' *E. coli* protein by the ribosome requires ≈9-15 seconds. Our previous results for tracking of the ribosomal subunits provide an estimate for an average time required for initiation for a ribosome at 1.3-1.6 s. Assuming that the termination of translation proceeds fast, we estimate that the average cycle time of the ribosome to translate a typical protein lies between 10-17 s.

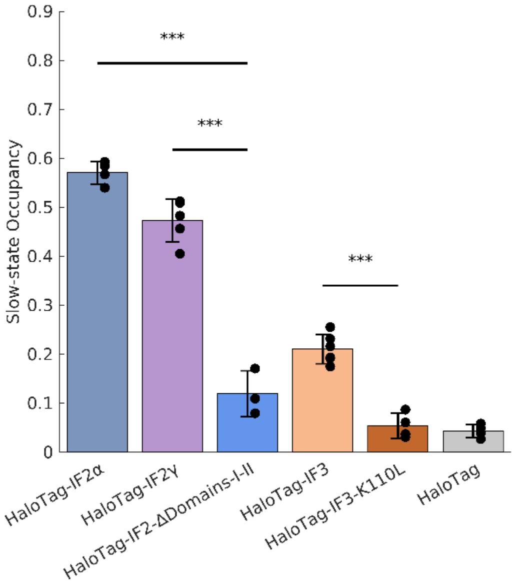

Supplementary Fig. 10. Effect of mutation on slow diffusion state occupancy of HaloTag-IF2 and HaloTag-IF3. Estimated steady-state occupancies of HaloTag-IF2α, HaloTag-IF2γ, variant of HaloTag-IF2 lacking Domain I and II, HaloTag-IF3, HaloTag-IF3-K110L mutant, and HaloTag in the slow diffusion state. The data show coarse-grained results from 5-state HMM models, with averages calculated from independent experiments. Error bars represent standard deviations between these independent experiments. The combined number of cells and trajectory steps were as follows: HaloTag-IF2α – 1,592 cells and 134,697 steps in 4 independent experiments; HaloTag-IF2γ – 1,622 cells and 127,210 steps in 5 independent experiments; HaloTag-IF2-ΔDomain-I-II – 1,470 cells and 125,190 steps in 3 independent experiments; HaloTag-IF3 – 2,491 cells and 172,989 steps in 6 independent experiments; HaloTag-IF3-K110L – 2,069 cells and 143,736 steps in 4 independent experiments; HaloTag – 2,630 cells and 109,253 steps in 4 independent experiments. Results for the 5-state models are provided in Supplementary Data 14-15. Results for all model sizes for combined data are provided in Supplementary Data 16-17. Statistical significance between groups was assessed using a two-sided unpaired t-test. P-values are indicated in the figure as follows: $P < 0.001$ (***). Source data are provided as a Source Data file.

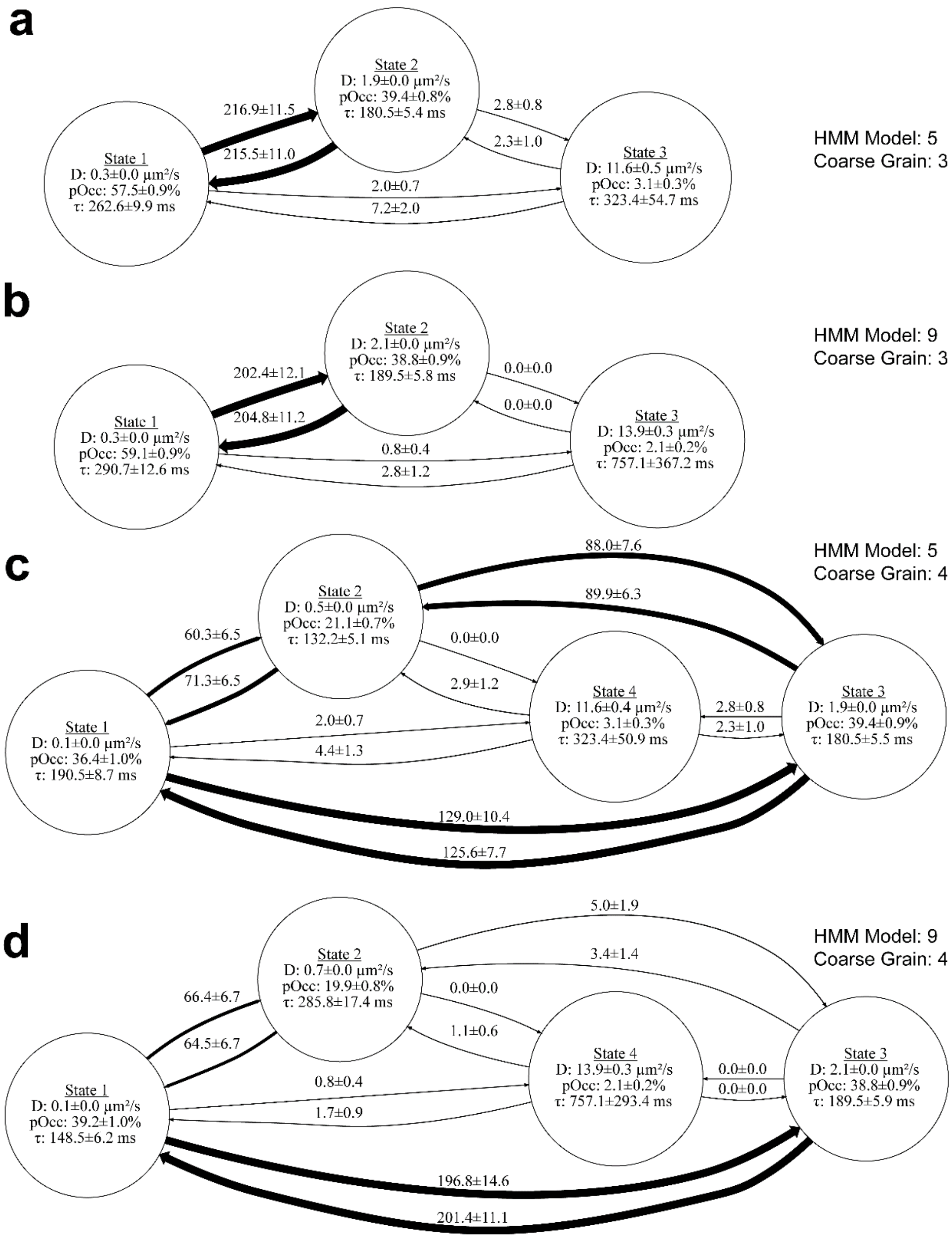

Supplementary Fig. 11. Fluxes of HaloTag-IF2α particles between states in HMM-fitted 5-state and 9-state models, coarse-grained to 3 or 4 diffusion states. Fluxes are shown for the 5-state (**a**, **c**) and 9-state (**b**, **d**) HMM models of HaloTag-IF2α, coarse-grained into either 3-state (**a**, **b**), or 4-state (**c**, **d**) models. In the 3-state models (**a**, **b**), coarse-graining was performed using diffusion thresholds at 1 μm²/s and 8 μm²/s to distinguish ribosome-bound IF2 (State 1), free IF2 (State 2), and cleavage products (State 3). In the 4-state models (**c**, **d**), thresholds at 0.25, 1 μm²/s, and 8

μm²/s were used to further differentiate "IF2–30S–mRNA" (State 1), "IF2–30S" (State 2), free IF2 (State 3), and cleavage products (State 4). Arrows indicate the direction of fluxes between coarse-grained states. Flux values between State X and State Y were calculated as the probability of transition between the two states per frame (5 ms), multiplied by the occupancy of State X, and scaled by a factor of 20,000 for visualization purposes. The fluxes are calculated for combined datasets for HaloTag-IF2α collected from 1,592 cells with 134,697 trajectory steps in 4 independent experiments. Errors for diffusion coefficients, occupancies and dwell times are bootstrap-estimated standard errors. For fluxes, the errors are propagated from the state occupancy and transition frequency bootstrap errors. Notably, fluxes in the 3-state coarse-grained models appear to be model-independent, whereas those in the 4-state models show model dependence, particularly for fluxes between State 2 and State 3, as well as between State 1 and State 3.

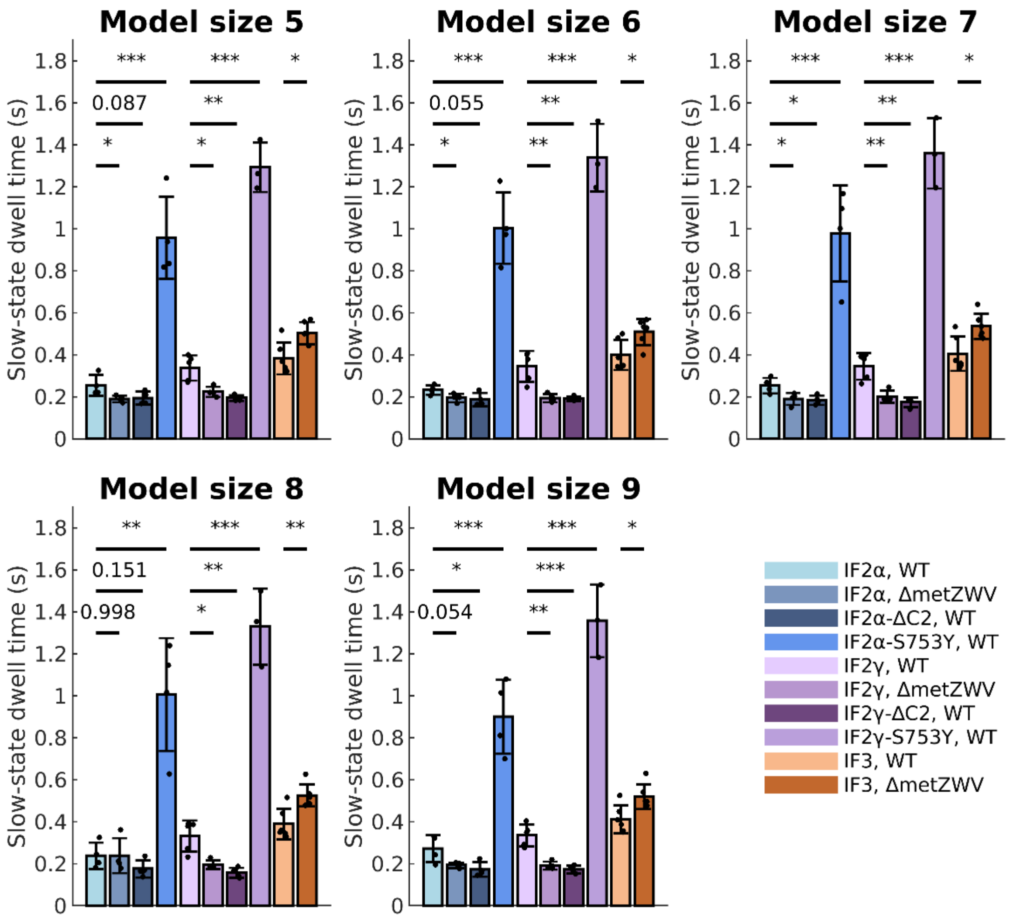

Supplementary Fig. 12 (related to Fig. 2c). Estimated dwell-times of HaloTag labeled IF3 as well as IF2 isoforms and mutants in the "bound" diffusion state from 3-state coarse-grained HMM models of sizes 5-9. In the *ΔmetZWV* strain, three of four genes for $tRNA^{fMet}$ are deleted, resulting in lower in vivo concentration of $fMet\text{-}tRNA^{fMet}$. The

data show results from 3-state coarse-grained HMM models of sizes 5-9, with averages calculated from independent experiments. Error bars represent standard deviations between these independent experiments. The combined number of cells and trajectory steps were as follows: HaloTag-IF2α – 1,592 cells and 134,697 steps in 4 independent experiments; HaloTag-IF2α in the *ΔmetZWV* strain – 1,399 cells and 110,116 steps in 4 independent experiments; HaloTag-IF2α-ΔC2 – 1,429 cells and 118,712 steps in 4 independent experiments; HaloTag-IF2α-S753Y – 1,825 cells and 131,921 steps in 4 independent experiments; HaloTag-IF2γ – 1,622 cells and 127,210 steps in 5 independent experiments; HaloTag-IF2γ in the *ΔmetZWV* strain – 1,609 cells and 127,467 steps in 4 independent experiments; HaloTag-IF2γ-ΔC2 – 1,606 cells and 140,172 steps in 4 independent experiments; HaloTag-IF2γ-S753Y – 1,727 cells and 139,119 steps in 3 independent experiments; HaloTag-IF3 – 2,491 cells and 172,989 steps in 6 independent experiments; and HaloTag-IF3 in *ΔmetZWV* – 2,919 cells and 246,146 steps in 6 independent experiments. Statistical significance between groups was assessed using a two-sided unpaired t-test. P-values are indicated in the figure as follows: $P < 0.05$ (*), $P < 0.01$ (**), $P < 0.001$ (***), P value is shown when $P > 0.05$. Source data are provided as a Source Data file.

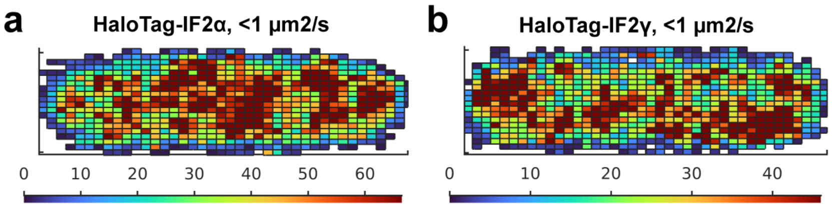

Supplementary Fig. 13. Spatial distribution of the slow diffusion state for HaloTag-IF2α and HaloTag-IF2γ isoforms. Spatial distribution of HaloTag-IF2α (**a**), HaloTag-IF2γ (**b**) in the slow diffusion state from 5-state HMM models coarse-grained into 3-states models. Size-based filtering was applied to exclude newly divided cells, characterized by a single nucleoid at mid-cell or two poorly separated lobes, as well as larger cells with more than two nucleoid lobes [7]. The combined number of cells and trajectory steps after cell sorting were as follows: HaloTag-IF2α – 590 cells and 53,233 trajectory steps in 4 independent experiments; HaloTag-IF2γ –572 cells and 46,113 trajectory steps in 5 independent experiments. Calculated occupancy in the slow state for HaloTag-IF2α and HaloTag-IF2γ, is 57% and 46%, respectively. Source data are provided as a Source Data file.

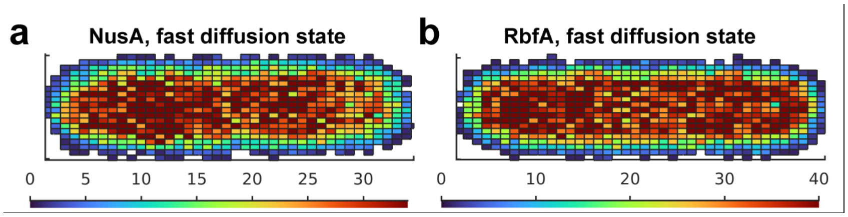

Supplementary Fig. 14 (related to Fig. 3). Spatial distribution of NusA-HaloTag and RbfA-HaloTag in the fast diffusion state. Spatial distribution of NusA-HaloTag (**a**), RbfA-HaloTag (**b**) in the fast diffusion state from 5-state HMM models coarse-grained into 2-states models using thresholds at 1 µm²/s and 1.2 µm²/s, respectively. A different threshold for RbfA-HaloTag was applied, as its main slow diffusion states exhibit diffusion coefficients in the range of 1–1.2 µm²/s. Size-based filtering was applied to exclude newly divided cells, characterized by a single nucleoid at mid-cell or two poorly separated lobes, as well as larger cells with more than two nucleoid lobes [7]. The combined number of cells and trajectory steps after cell sorting were as follows: NusA-HaloTag – 762 cells and 71,823 trajectory steps in 3 independent experiments; RbfA-HaloTag – 686 cells and 39,683 trajectory steps in 7 independent experiments. Calculated occupancy in the fast state for NusA-HaloTag and RbfA-HaloTag, is 22% and 50%, respectively. Source data are provided as a Source Data file.

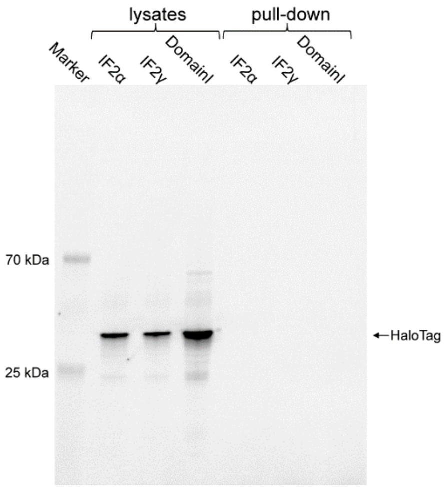

Supplementary Fig. 15. SDS-PAGE analysis of cell lysates and affinity purification fractions for the presence of HaloTag. 6His-IF2α, 6His-IF2γ, and IF2-Domain-I-6His were overexpressed in an *E. coli* strain carrying a plasmid for expression of HaloTag. Lysates and samples affinity purified on Co-NTA agarose (i.e. “pull-down”) were loaded on SDS-PAGE gel and stained using the JFX549 dye to label the HaloTag. Source data are provided as a Source Data file.

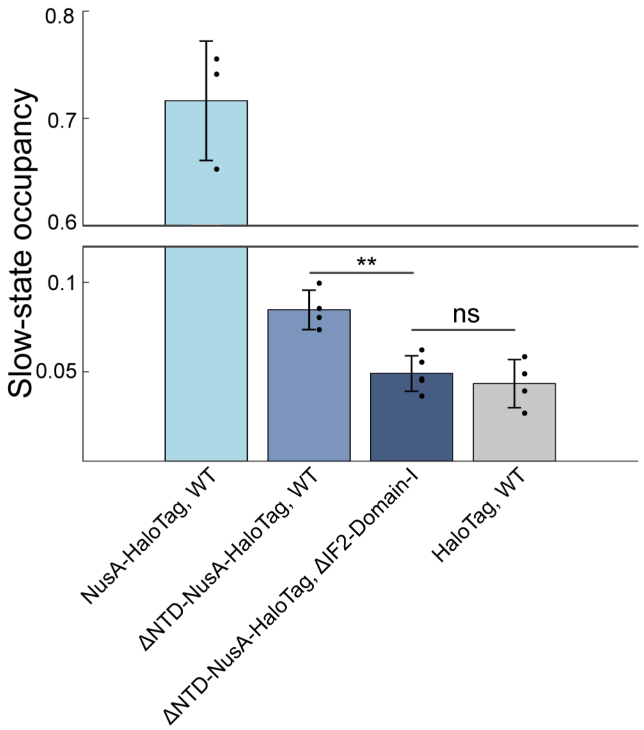

Supplementary Fig. 16. Effect of NTD deletion on slow diffusion state occupancy of NusA-HaloTag in WT and in E. coli ΔIF2-Domain-I. Estimated steady-state occupancies of NusA-HaloTag, and ΔNTD-NusA-HaloTag, and HaloTag in WT and in *E. coli* ΔIF2-Domain-I strains in the slow diffusion state. The data show results from 5-state HMM models coarse grained into 2-state models using threshold at 1 $\mu m^2/s$, with averages calculated from independent experiments. Error bars represent standard deviations between these independent experiments. The combined number of cells and trajectory steps were as follows: NusA-HaloTag in WT strain – 1,996 cells and 170,214 trajectory steps in 3 independent experiments; ΔNTD-NusA-HaloTag in WT strain – 906 cells and 50,803 trajectory steps in 4 independent experiments; ΔNTD-NusA-HaloTag in in *E. coli* ΔIF2-Domain-I strain – 2,238 cells and 120,766 trajectory steps in 5 independent experiments; HaloTag – 2,630 cells and 109,253 trajectory steps in 4 independent experiments. Results for the 5-state models are provided in Supplementary Data 45, 48. Results for all model sizes for combined data are provided in Supplementary Data 47, 50. Statistical significance between groups was assessed using a two-sided unpaired t-test. P-values are indicated in the figure as follows: $P < 0.01$ (**), and not significant (ns) otherwise. Source data are provided as a Source Data file.

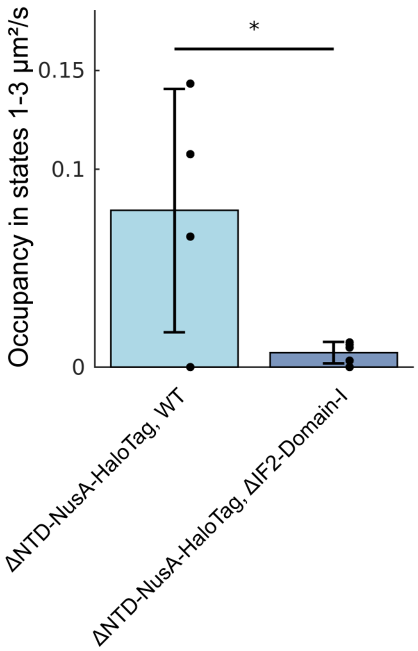

Supplementary Fig. 17. Interaction between NusA and IF2α in the bacterial cytoplasm. Estimated steady-state occupancies of ΔNTD-NusA-HaloTag in WT and in *E. coli* ΔIF2-Domain-I strains in the diffusion states from 1 μm²/s to 3 μm²/s, characteristic for fast diffusion state of IF2α. The data show results from 9-state HMM models coarse grained into 3-state models using threshold at 1 μm²/s and 3 μm²/s, with averages calculated from independent experiments. Error bars represent standard deviations between these independent experiments. The combined number of cells and trajectory steps were as follows: ΔNTD-NusA-HaloTag in WT strain – 906 cells and 50,803 trajectory steps in 4 independent experiments; ΔNTD-NusA-HaloTag in in *E. coli* ΔIF2-Domain-I strain – 2,238 cells and 120,766 trajectory steps in 5 independent experiments. Results for the 9-state models for individual experiments are provided in Supplementary Data 46, 49. Results for all model sizes for combined data are provided in Supplementary Data 47, 50. Statistical significance between groups was assessed using a two-sided unpaired t-test. P-values are indicated in the figure as follows: $P < 0.05$ (*). Source data are provided as a Source Data file.

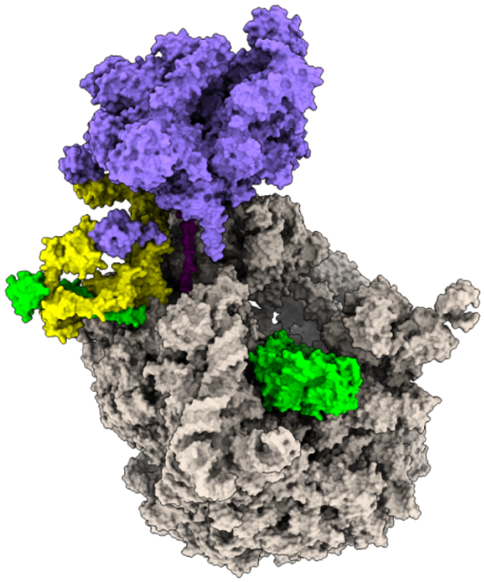

Supplementary Fig. 18. Modelling of a coupled transcription-translation complex with fitted IF2. Structure of a coupled transcription-translation complex containing NusA and NusG (6X7F PDB) was aligned with the structure of ribosome-bound IF2 (3JCJ PDB) to model the position of the C-terminal part of IF2 (amino acids 382-890) and aligned with the Alphafold-modeled complex between NusA and N-terminal fragment of IF2 (amino acids 2-94). Both fitted fragments of IF2 are shown in green, NusA is shown in yellow, RNAP is shown in purple, ribosome is shown in grey.

REFERENCES

1. Antoun A, Pavlov MY, Lovmar M, Ehrenberg M. How initiation factors maximize the accuracy of tRNA selection in initiation of bacterial protein synthesis. *Mol Cell* **23**, 183-193 (2006).

2. Dai X*, et al.* Reduction of translating ribosomes enables Escherichia coli to maintain elongation rates during slow growth. *Nat Microbiol* **2**, 16231 (2016).

3. Bremer H, Dennis PP. Modulation of Chemical Composition and Other Parameters of the Cell at Different Exponential Growth Rates. *EcoSal Plus* **3**, (2008).

4. Li GW, Burkhardt D, Gross C, Weissman JS. Quantifying absolute protein synthesis rates reveals principles underlying allocation of cellular resources. *Cell* **157**, 624-635 (2014).

5. Metelev M, Lundin E, Volkov IL, Gynnå AH, Elf J, Johansson M. Direct measurements of mRNA translation kinetics in living cells. *Nat Commun* **13**, (2022).

6. Schmidt A*, et al.* The quantitative and condition-dependent Escherichia coli proteome. *Nat Biotechnol* **34**, 104-110 (2016).

7. Bakshi S, Siryaporn A, Goulian M, Weisshaar JC. Superresolution imaging of ribosomes and RNA polymerase in live Escherichia coli cells. *Mol Microbiol* **85**, 21-38 (2012).
