# Supplementary material for: A complex between IF2 and NusA suggests early coupling of transcription-translation
Source: Nat Commun. 2025 Jul 26;16:6906. doi: 10.1038/s41467-025-62207-w (PMC12297416; doi:10.1038/s41467-025-62207-w)
Supplement: Supplementary file 2 — Description of Additional Supplementary Files [file 41467_2025_62207_MOESM2_ESM.pdf]

Description of Additional Supplementary Files

Supplementary Data 1: Results of HMM analysis for tracking of HaloTag-IF2α isoform, combined data.

Supplementary Data 2: Results of HMM analysis for tracking of HaloTag-IF2γ isoform, combined data.

Supplementary Data 3: Results of HMM analysis for tracking of HaloTag-IF3, combined data.

Supplementary Data 4: Results of HMM analysis for tracking of HaloTag-IF1, combined data.

Supplementary Data 5: Results of HMM analysis for tracking of fMet-tRNAfMet-Cy5, combined data.

Supplementary Data 6: Results of HMM analysis for tracking of 30S ribosomal subunits labeled using h6 aptamer and MS2CP-HaloTag, combined data.

Supplementary Data 7: Results of HMM analysis for tracking of free HaloTag, combined data.

Supplementary Data 8: Results of HMM analysis for tracking of HaloTag-IF2α isoform, individual datasets for 5-state models.

Supplementary Data 9: Results of HMM analysis for tracking of HaloTag-IF2γ isoform, individual datasets for 5-state models.

Supplementary Data 10: Results of HMM analysis for tracking of HaloTag-IF3, individual datasets for 5-state models.

Supplementary Data 11: Results of HMM analysis for tracking of HaloTag-IF1, individual datasets for 5-state models.

Supplementary Data 12: Results of HMM analysis for tracking of fMet-tRNAfMet-Cy5, individual datasets for 5-state models.

Supplementary Data 13: Results of HMM analysis for tracking of free HaloTag, individual datasets for 5-state models.

Supplementary Data 14: Results of HMM analysis for tracking of HaloTag-IF2 variant lacking Domain I and II, individual datasets for 5-state models.

Supplementary Data 15: Results of HMM analysis for tracking of HaloTag-IF3 mutant with the K110L substitution, individual datasets for 5-state models.

Supplementary Data 16: Results of HMM analysis for tracking of HaloTag-IF2 variant lacking Domain I and II, combined data.

Supplementary Data 17: Results of HMM analysis for tracking of HaloTag-IF3 mutant with the K110L substitution, combined data.

Supplementary Data 18: Results of HMM analysis for tracking of HaloTag-IF2α isoform in E. coli ΔMetZWV strain, individual datasets for 5-state models.

Supplementary Data 19: Results of HMM analysis for tracking of HaloTag-IF2γ isoform in E. coli ΔMetZWV strain, individual datasets for 5-state models.

Supplementary Data 20: Results of HMM analysis for tracking of HaloTag-IF2α mutant with deletion of the Domain VI-2, individual datasets for 5-state models.

Supplementary Data 21: Results of HMM analysis for tracking of HaloTag-IF2γ mutant with deletion of the Domain VI-2, individual datasets for 5-state models.

Supplementary Data 22: Results of HMM analysis for tracking of HaloTag-IF2α mutant with substitution S753Y, individual datasets for 5-state models.

Supplementary Data 23: Results of HMM analysis for tracking of HaloTag-IF2γ mutant with substitution S753Y, individual datasets for 5-state models.

Supplementary Data 24: Results of HMM analysis for tracking of HaloTag-IF3 isoform in E. coli ΔMetZWV strain, individual datasets for 5-state models.

Supplementary Data 25: Results of HMM analysis for tracking of HaloTag-IF2α isoform in E. coli ΔMetZWV strain, combined data.

Supplementary Data 26: Results of HMM analysis for tracking of HaloTag-IF2γ isoform in E. coli ΔMetZWV strain, combined data.

Supplementary Data 27: Results of HMM analysis for tracking of HaloTag-IF2α mutant with deletion of the Domain VI-2, combined data.

Supplementary Data 28: Results of HMM analysis for tracking of HaloTag-IF2γ mutant with deletion of the Domain VI-2, combined data.

Supplementary Data 29: Results of HMM analysis for tracking of HaloTag-IF2α mutant with substitution S753Y, combined data.

Supplementary Data 30: Results of HMM analysis for tracking of HaloTag-IF2γ mutant with substitution S753Y, combined data.

Supplementary Data 31: Results of HMM analysis for tracking of HaloTag-IF3 isoform in E. coli ΔMetZWV strain, combined data.

Supplementary Data 32: Results of HMM analysis for tracking of IF2-Domain-I-HaloTag, combined data.

Supplementary Data 33: Results of HMM analysis for tracking of IF2-Domain-I-HaloTag, individual datasets for 5-state models.

Supplementary Data 34: Results of HMM analysis for tracking of RpoC-HaloTag, combined data.

Supplementary Data 35: Results of HMM analysis for tracking of NusA-HaloTag, individual datasets for 5-state models.

Supplementary Data 36: Results of HMM analysis for tracking of NusA-HaloTag, combined data.

Supplementary Data 37: Results of HMM analysis for tracking of RbfA-HaloTag, individual datasets for 5-state models.

Supplementary Data 38: Results of HMM analysis for tracking of RbfA-HaloTag, combined data.

Supplementary Data 39: Results of HMM analysis for tracking of IF2-Domain-I-HaloTag in E. coli NusA-ΔC strain, individual datasets for 5-state models.

Supplementary Data 40: Results of HMM analysis for tracking of IF2-Domain-I-HaloTag in E. coli NusA-ΔC strain with pNusA plasmid without induction, individual datasets for 5-state models.

Supplementary Data 41: Results of HMM analysis for tracking of IF2-Domain-I-HaloTag in E. coli NusA-ΔC strain with pNusA plasmid with induction, individual datasets for 5-state models.

Supplementary Data 42: Results of HMM analysis for tracking of IF2-Domain-I-HaloTag in E. coli NusA-ΔC strain, combined data.

Supplementary Data 43: Results of HMM analysis for tracking of IF2-Domain-I-HaloTag in E. coli NusA-ΔC strain with pNusA plasmid without induction, combined data.

Supplementary Data 44: Results of HMM analysis for tracking of IF2-Domain-I-HaloTag in E. coli NusA-ΔC strain with pNusA plasmid with induction, combined data.

Supplementary Data 45: Results of HMM analysis for tracking of dNTD-NusA-HaloTag in ΔIF2-Domain-I strain, individual datasets for 5-state models.

Supplementary Data 46: Results of HMM analysis for tracking of dNTD-NusA-HaloTag in ΔIF2-Domain-I strain, individual datasets for 9-state models.

Supplementary Data 47: Results of HMM analysis for tracking of dNTD-NusA-HaloTag in ΔIF2-Domain-I strain, combined data.

Supplementary Data 48: Results of HMM analysis for tracking of dNTD-NusA-HaloTag in WT strain, individual datasets for 5-state models.

Supplementary Data 49: Results of HMM analysis for tracking of dNTD-NusA-HaloTag in WT strain, individual datasets for 9-state models.

Supplementary Data 50: Results of HMM analysis for tracking of dNTD-NusA-HaloTag in WT strain, combined data.

Supplementary Data 51: Results of HMM analysis for tracking of HaloTag-IF2α-S753Y in E. coli ΔIF2-Domain-I strain strain, < 1 µm2/s, combined data.

Supplementary Data 52: Results of HMM analysis for tracking of HaloTag-IF2α-S753Y in E. coli ΔIF2-Domain-I strain strain, < 0.25 µm2/s, combined data.

Supplementary Data 53: Results of HMM analysis for tracking of HaloTag-IF2α-S753Y in E. coli ΔIF2-Domain-I strain strain, < 0.05 µm2/s, combined data.

Supplementary Data 54: Results of HMM analysis for tracking of HaloTag-IF2γ-S753Y in E. coli ΔIF2-Domain-I strain strain, < 1 µm2/s, combined data.

Supplementary Data 55: Results of HMM analysis for tracking of HaloTag-IF2γ-S753Y in E. coli ΔIF2-Domain-I strain strain, < 0.25 µm2/s, combined data.

Supplementary Data 56: Results of HMM analysis for tracking of HaloTag-IF2γ-S753Y in E. coli ΔIF2-Domain-I strain strain, < 0.05 µm2/s, combined data.

Supplementary Data 57: Oligonucleotides.

Supplementary Data 58: Constructed plasmids.

Supplementary Data 59: Constructed E. coli strains.

Supplementary Movie 1: Microscopy data of HaloTag-IF1 diffusion in live E. coli. The movie (top panel) was acquired with 5 ms camera exposure time and 3 ms laser illumination (546 nm) per image. For analysis, movies were aligned with cell outlines (segmented based on phase contrast images, middle panel), and trajectories of single HaloTag-IF1 particles were built using the uTrack algorithm and HMM-fitted to a 2-state diffusion model with state 1 corresponding to slow diffusion and state 2 to fast diffusion (bottom panel). To reduce the risk of errors in the trajectory building, trajectories were recorded when there was only one fluorescent dot detected in a cell. Playback speed is 15 frames per second.

Supplementary Movie 2: Microscopy data of HaloTag-IF2α diffusion in live E. coli. The movie (top panel) was acquired with 5 ms camera exposure time and 3 ms laser illumination (546 nm) per image. For analysis, movies were aligned with cell outlines (segmented based on phase contrast images, middle panel), and trajectories of single HaloTag-IF2α particles were built using the uTrack algorithm and HMM-fitted to a 2-state diffusion model with state 1 corresponding to slow diffusion and state 2 to fast diffusion (bottom panel). To reduce the risk of errors in the trajectory building, trajectories were recorded when there was only one fluorescent dot detected in a cell. Playback speed is 15 frames per second.

Supplementary Movie 3: Microscopy data of HaloTag-IF2γ diffusion in live E. coli. The movie (top panel) was acquired with 5 ms camera exposuretime and 3 ms laser illumination (546 nm) per image. For analysis, movies were aligned with cell outlines (segmented based on phase contrast images, middle panel), and trajectories of single HaloTag-IF2γ particles were built using the uTrack algorithm and HMM-fitted to a 2-state diffusion model with state 1 corresponding to slow diffusion and state 2 to fast diffusion (bottom panel). To reduce the risk of errors in the trajectory building, trajectories were recorded when there was only one fluorescent dot detected in a cell. Playback speed is 15 frames per second.

Supplementary Movie 4: Microscopy data of HaloTag-IF3 diffusion in live E. coli. The movie (top panel) was acquired with 5 ms camera exposure time and 3 ms laser illumination (546 nm) per image. For analysis, movies were aligned with cell outlines (segmented based on phase contrast images, middle panel), and trajectories of single HaloTag-IF3 particles were built using the uTrack algorithm and HMM-fitted to a 2-state diffusion model with state 1 corresponding to slow diffusion and state 2 to fast diffusion (bottom panel). To reduce the risk of errors in the trajectory building, trajectories were recorded when there was only one fluorescent dot detected in a cell. Playback speed is 15 frames per second.

Supplementary Movie 5: Microscopy data of fMet-[Cy5]tRNAfMet diffusion in live E. coli. The movie (left panel) was acquired with 5 ms camera exposure time and 1.5 ms laser illumination (642 nm) per image. For analysis, movies were aligned with cell outlines (segmented based on phase contrast images, middle panel), and trajectories of single fMet-[Cy5]tRNAfMet particles were built using the uTrack algorithm and HMM-fitted to a 2-state diffusion model with state 1 corresponding to slow diffusion and state 2 to fast diffusion (right panel). To reduce the risk of errors in the trajectory

building, trajectories were recorded when there was only one fluorescent dot detected in a cell. Playback speed is 15 frames per second.

Supplementary Movie 6: Microscopy data of 30S-HaloTag diffusion in live E. coli. The movie (top panel) was acquired with 5 ms camera exposure time and 3 ms laser illumination (546 nm) per image. For analysis, movies were aligned with cell outlines (segmented based on phase contrast images, middle panel), and trajectories of single 30S-HaloTag particles were built using the uTrack algorithm and HMM-fitted to a 2-state diffusion model with state 1 corresponding to slow diffusion and state 2 to fast diffusion (bottom panel). To reduce the risk of errors in the trajectory building, trajectories were recorded when there was only one fluorescent dot detected in a cell. Playback speed is 15 frames per second.
